# Adult validation of a self-administered tablet audiometer

**DOI:** 10.1186/s40463-019-0385-0

**Published:** 2019-11-07

**Authors:** Mark Bastianelli, Amy E. Mark, Arran McAfee, David Schramm, Renée Lefrançois, Matthew Bromwich

**Affiliations:** 1 Faculty of Medicine, University of Ottawa, Ottawa, Ontario Canada; 2 Department of Otolaryngology – Head and Neck Surgery, The Ottawa Hospital, Ottawa, Ontario Canada; 3 Department of Otolaryngology – Head and Neck Surgery, University of Ottawa, Ottawa, Ontario Canada; 4 Division of Pediatric Otolaryngology – Head and Neck Surgery, Children’s Hospital of Eastern Ontario, Ottawa, Ontario Canada; 5 Children’s Hospital of Easter Ontario Research Institute, Ottawa, Canada; 6 SHOEBOX Inc, Ottawa, Ontario Canada; 7 Ottawa Hospital Research Institute, Ottawa, Ontario Canada

**Keywords:** Automated audiometry, Audiology, Tablet audiometry, Screening audiometry, Hearing loss

## Abstract

**Background:**

There is evidence to suggest that rates of hearing loss are increasing more rapidly than the capacity of traditional audiometry resources for screening. A novel innovation in tablet, self-administered portable audiometry has been proposed as a solution to this discordance. The primary objective of this study was to validate a tablet audiometer with adult patients in a clinical setting. Secondarily, word recognition with a tablet audiometer was compared against conventional audiometry.

**Methods:**

Three distinct prospective adult cohorts underwent testing. In group 1 and group 2 testing with the automated tablet audiometer was compared to standard sound booth audiometry. In Group 1, participants’ pure tone thresholds were measured with an automated tablet audiometer in a quiet clinic exam room. In Group 2, participants completed monosyllabic word recognition testing using the NU-6 word lists. In Group 3, internal reliability was tested by having participants perform two automated tablet audiometric evaluation in sequence.

**Results:**

Group 1 included 40 patients mean age was 54.7 ± 18.4 years old and 60% female; Group 2 included 44 participants mean age was 55.2 ± 14.8 years old and 68.2% female; Group 3 included 40 participants with mean age of 39.4 + 15.9 years old and 60.5% female. In Group 1, compared to standard audiometry, 95.7% (95% CI: 92.6–98.9%) of thresholds were within 10 dB. In Group 2, comparing word recognition results, 96.2% (95% CI: 89.5–98.7%) were clinically equivalent and within a critical difference range. In Group 3, One-way Intraclass Correlation for agreement for the both left- and right-ear pure tone average was 0.98. The mean difference between repeat assessments was 0 (SD = 2.1) in the left ear, and 0.1 (SD = 1.1) in the right ear.

**Conclusion:**

Puretone audiometry and word recognition testing appears valid when performed by non-healthcare experts using a tablet audiometer outside a sound booth in a quiet environment.

**Trial registration:**

ClinicalTrials.gov Identifier: NCT02761798.

Registered April, 2016 < https://clinicaltrials.gov/ct2/show/NCT02761798>

## Background

In the 2016 report on hearing loss based on the Canadian Health Measures Survey audiometry results from 2012 to 2015 indicated that 40% of Canadian adults between 20 and 79 years of age had at least mild hearing loss in at least one ear [1]. Furthermore, 77% of these adults with measured hearing loss were unaware of their pre-existing diagnosis. With aging being the number one cause of hearing loss, the incidence of hearing loss is projected to rise dramatically. The number of adults aged 65 and over in Canada is expected to double to 9.9–10.9 million people by 2036 [2]. This poses a significant challenge in order to provide both timely and cost-effective access for these patients to audiometric services.

It is well established that undiagnosed/untreated hearing loss can lead to physiological changes associated with auditory deprivation, as well as psychosocial changes of social isolation and depression [3, 4]. Hearing loss can result in emotional, physical, cognitive, and behavioral consequences including impaired activities of daily living, decline in independence and reduced quality of life [5–8]. Early identification can help to reduce these deleterious effects, and lead to easier adjustment to hearing aid use [9, 10].

Conventionally, audiometric testing for adults is performed by a trained audiologist in a sound-insulated booth. However access to hearing healthcare has been shown to be limited based on several factors including geographic location, provider variables (access to specialist or primary care services) and socioeconomic status [11, 12]. Current guidance from the American Speech-Language-Hearing Association (ASHA) recommends that adults be screened every 10 years through from age 19–50 and every 3 years thereafter and yearly in patients with identified hearing loss [13]. In one study where 4556 American adults were surveyed, 65% of respondents reported having a hearing test greater than 10 years ago or never tested at all [14]. This emphasizes the crucial need for improvement of the current model for hearing health care in North America in order to provide patients with both timely as well as effective audiometric screening.

One emerging solution to this public health problem is to utilize automated audiometry for screening when conventional audiometry is not available. Automated audiometry uses a computer-based software as well as the standard protocols that are used by audiologists to perform both air and bone conduction hearing testing. Several reports have previously demonstrated that this method is effective and reliable for screening in both children and adults [15–18]. One of the major benefits of automated audiometry is its potential applications in situations or locations where audiologists or sound insulated booths are unavailable. Tablet devices present a unique platform to utilize the automated audiometric technology in a portable fashion.

A portable automated audiometer for the Apple iOS platform (SHOEBOX™ Audiometry, SHOEBOX Inc., Ottawa, ON), has previously been described and utilized in a variety of clinical scenarios [19–23]. This method of testing provides the tools to non-hearing healthcare experts to facilitate point of care evaluation, where the patient seeks the sound stimulus through an interactive and intuitive platform.

Previously published studies were completed on earlier versions of the software and did not evaluate the internal reliability or word recognition testing modality. Therefore, the objective of this multi-group study was to evaluate the capability of the tablet audiometer and record its performance when used in an adult patient population. In Group 1, the performance and the capability to measure hearing loss of the tablet audiometer was compared to conventional audiometry. In Group 2, word recognition testing using the tablet audiometer was compared against conventional audiometry. Finally, in Group 3, the internal reliability of the tablet audiometer was evaluated using a test-retest methodology.

## Methodology

This study was approved by The Ottawa Health Science Network Research Ethics Board (OHSN-REB #20150561-01H). The tablet audiometer meets ANSI/ASA S3.6–2010 requirements for audiometry, and is registered with Health Canada and the FDA as a Class II Medical Device for adults and children. Calibration of the tablet-audiometry transducers are completed every 12 months or sooner in accordance with the ANSI/ASA S3.6–2010 Specifications for Audiometers guidelines.

This study was administered in three separate groups of participants between May 2016 and October 2017. For all groups, adults over the age of 18 years who attended the Otolaryngology/Audiology clinic at The Ottawa Hospital were eligible to participate. Potential participants were excluded if they self-reported as being unable for any reason to use a tablet device or in Group 2, anyone who had masking during word recognition testing in the sound booth. Group 2 participants were also limited to English speaking adults.

The Clinical Research Coordinator (CRC) was present to explain the study, answer questions and obtain informed consent. Eligible patients who provided consent were enrolled in the study. During the execution of the automated tablet audiometric testing, the CRC remained present to answer questions, observe behavior, and anecdotally record participant feedback while the participant completed the automated audiometric test. The CRC has a nursing background but has not received any formal audiological training.

Conventional audiometric evaluation was carried out by audiologists in a double walled sound booth using a GSI-61 Audiometer and followed standard protocols outlined in the “Practice Standards and Guidelines for Hearing Assessment of Adults by Audiologists,” from the College of Audiologists and Speech-Language Pathologists of Ontario (CASLPO) [24].

In Groups 1 and 3, hearing testing with the tablet audiometer was performed; pure tone air conduction thresholds were obtained through the automated game platform. The software employs a modified Hughson-Westlake protocol with a two-alternative force choice paradigm (i.e. participant sorts an object based on whether or not a sound is heard). All participants were tested at frequencies of 250, 500, 1000, 2000, 4000, and 8000 Hz using calibrated ER3A audiometric insert transducers. Lower and upper tone presentation volumes were limited at 10 dB and 90 dB, respectively. Masking using the tablet audiometer occurs automatically in accordance with Katz et al. methods of clinical masking that have been previously described [25].

### Group 1

Each participant underwent conventional pure tone audiometric testing administered by an audiologist followed by an automated tablet audiometric test. The tablet audiometric evaluations were performed in a quiet but non sound-insulated clinical exam room. Both tests were conducted using conventional ER3A inserts transducers on the same day.

### Group 2

Participants underwent word recognition testing using both conventional audiometry as well as tablet audiometry. Word recognition was performed by way of the Northwestern University Auditory Test # 6 (NU-6, Form A Lists 1–4, Auditec Inc). In view that the recorded NU-6 word list is available in English only, participants required a working understanding of English in order to participate in Group 2 of this study.

Patients first underwent conventional audiometry including word recognition testing performed by an Audiologist in a sound insulated booth. The Audiologist presented recorded NU-6 lists 1a and 1b (50 words) using calibrated ER3A inserts at the patient’s most comfortable listening level (MCL). Pre-recorded word lists were used rather than live voice during traditional audiometry to enhance standardization.

Next, the tablet audiometer was manually configured to perform the word recognition testing using the same MCL that was determined by the Audiologist during the patient audiogram performed in the sound booth. Word recognition testing by the tablet audiometer was performed using calibrated ER3A inserts in a non-sound-insulated clinical exam room. The word was presented to the patient through the insert transducers, the patient then repeated the word to the CRC who recorded the patient’s accuracy on the tablet audiometer. During the tablet word recognition testing, NU-6 2a and 2b word lists (50 words) were used in order to minimize the risk of recall by the participants between the two tests.

### Group 3

Participants self-administered the tablet audiogram twice in sequence using calibrated TDH-50 supra-aural headphones in a quiet, but non sound-insulated clinical exam room. The first test was performed and this was immediately followed by a second repeat tablet audiometric evaluation. The headphones were neither removed nor repositioned between tests.

## Statistical analysis

### Group 1

Clinically relevant hearing loss was, for the purposes of this analysis, defined as at least one threshold at or above 40 dB HL for either ear. As a sensitivity analysis, a threshold of 30 dB HL was also evaluated. 95% confidence intervals for sensitivity and specificity were computed using the Wilson score method [26].

Agreement between traditional audiometry and tablet audiometry was evaluated using Cohen’s unweighted kappa (for absolute agreement) and Cohen’s weighted kappa (to take the extent of disagreement into account).

The percentage of agreements within 10 dB and within 5 dB were calculated with 95% Wilson-score confidence intervals. The mean percentage agreement across all frequencies was computed with 95% confidence intervals derived from Student’s t-distribution. We conducted the analysis with and without frequencies of 250 Hz and 8000 Hz as measurements at these two frequencies have been known to be affected by background noise and transducer placement and tinnitus [27].

### Group 2

To evaluate for significant differences in computed word recognition scores we used the critical difference range that has previously been well described for comparing consecutive word recognition testing [28]. The mean difference between tablet and conventional audiometric assessments for each patient was obtained to further characterize the performance of the tablet audiometer to perform word recognition testing. In their 1978 paper, Thornton and Raffin determined that with a 25 set word list there are wide ranges in percent scores that are in reality clinically irrelevant. These critical difference ranges narrow somewhat with a 50 word set. For the purposes of this study actual percent scores were classified using the critical difference ranges for both methods of testing. Percent agreement was determined along with 95% confidence intervals.

### Group 3

A mixed effects model was used to determine if the thresholds obtained with the two tablet tests were significantly different from each other. One-way intraclass correlation coefficient (ICC) was computed to determine the internal reliability. The mean difference between repeat assessments for each patient was obtained to further characterize the nature of the reliability. ICC was selected because – unlike Pearson correlation – it can detect systematic absolute differences between repeat assessments**.**

## Results

### Group 1

A total of 40 patients were included in this group. The mean age was 54.7 years old and 60% patients were female (Table 1). Hearing levels for patients in this group are detailed in Table 8.

**Table 1:** – Demographic data for Group 1

Demographic characteristics	*N* = 40
Age (years); mean (SD)	54.7 (18.4)
Age (years); median (range)	53.8 (20.3–86.9)
Female; *n* (%)	24/40 (60.0)
Proportion of participants with two ears assessed; n (%)	39/40 (97.5)

When we compared the measured pure tone thresholds computed using the tablet audiometer to standard audiometry, we found that 92.9% of thresholds at all frequencies were within 10 dB. When we excluded frequencies of 250 Hz and 8000 Hz, 95.7% of thresholds were within 10 dB and 84.9% of thresholds were within 5 dB (Tables 2 and 3).

**Table 2:** – Percentage agreement (within 10 dB) between tablet audiometer and conventional audiometer at frequencies of 250 Hz, 500 Hz, 1000, 2000 Hz, 4000 Hz, and 8000 Hz

	250 Hz	500 Hz	1000 Hz	2000 Hz	4000 Hz	8000 Hz	mean	mean excluding AC250 Hz and AC8000 Hz
Percent Agreement	85.9	91.8	97.1	96.9	100.0	85.5	92.9	95.7
95% low	76.0	83.2	89.9	89.5	94.0	74.7	88.8	92.6
95% High	92.2	96.2	99.2	99.2	100.0	92.2	97.6	98.9

**Table 3:** – Percentage agreement (within 5 dB) between tablet audiometer and conventional audiometer at frequencies of 250 Hz, 500 Hz, 1000, 2000 Hz, 4000 Hz, and 8000 Hz

	250 Hz	500 Hz	1000 Hz	2000 Hz	4000 Hz	8000 Hz	mean	mean excluding 250 Hz and 8000 Hz
Percent Agreement	61.6	75.7	85.7	90.9	93.4	65.1	78.7	84.9
95% low	50.2	64.8	75.7	81.6	84.3	52.8	69.9	77.7
95% High	71.9	84.0	92.1	95.8	97.4	75.7	89.2	92.1

A sensitivity and specificity analysis are shown in Tables 4 and 5. When hearing loss was defined as having at least one threshold of 40 dB HL or greater in at least one ear the sensitivity and specificity was calculated to be 96 and 100% respectively (Table 4). When hearing loss was defined as having at least one threshold of 30 dB HL or greater in at least one ear the sensitivity was 100% with a specificity of 91% (Table 5).

**Table 4:** – 2 × 2 contingency table comparison of tablet audiometry to conventional audiometry when hearing loss defined as at least one threshold of 40 dB HL or greater in at least one ear

	Traditional Audiometry	
Tablet audiometry (SHOEBOX)	Normal hearing	Hearing loss
Normal hearing	13	1
Hearing loss	0	25
Sensitivity: 96% (95% CI 81, 99%), Specificity: 100% (95% CI 77, 100%)

**Table 5:** – 2 × 2 contingency table comparison of tablet audiometry to conventional audiometry when hearing loss defined as at least one threshold of 30 dB HL or greater in at least one ear

	Traditional Audiometry	
Tablet audiometry (SHOEBOX)	Normal hearing	Hearing loss
Normal hearing	10	0
Hearing loss	1	28
Sensitivity: 100% (95% CI 88, 100%), Specificity: 91% (95% CI 62, 98%)		

### Group 2

In Group 2 of this trial we recruited 44 patients. The average age was 55.2 and 68.2% of patient population was female (Table 6)**.**

**Table 6:** – Demographic data for Group 2

Demographic characteristics	*N* = 44
Age, mean (SD)	55.2 (14.8)
Age, median (IQR)	55.6 (44.7, 64.9)
Female, *n* (%)	30 (68.2)
participants with two ears assessed, n (%)	39 (88.6)

**Fig. 1:**
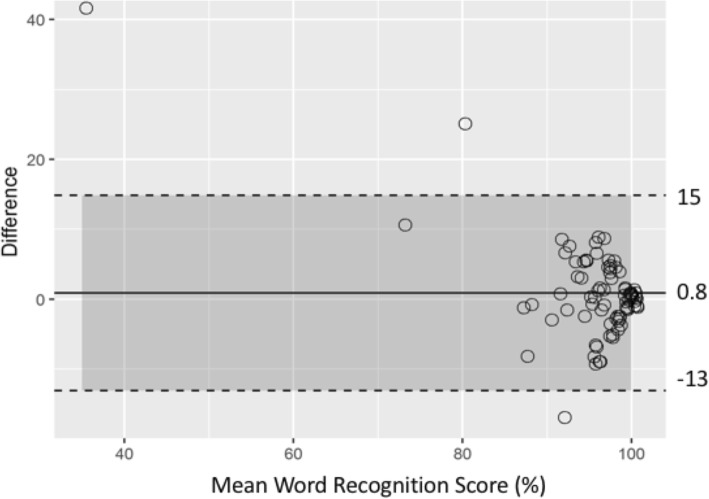
Group 2 Bland Altman Plot – Inter-score difference for word recognition scores between tablet and conventional assessments. Dotted lines depict 95% limits of agreement. Mean difference of 0.8 (95% CI 15,-13)

When we compared the word recognition scores using conventional audiometry and the tablet audiometer we determined that 96.2% (95% CI 89.5, 98.7%) of word recognition scores fell within the critical difference range and could therefore be considered equivalent. Bland-Altman graph is depicted in Fig. 1.

### Group 3

40 patients were enrolled in the sub-study. Two patients were excluded for declining to complete both tablet audiogram tests. The mean age of the patient population was 40 and 60.5% of patients were female (Table 7). Hearing levels for patients in this group are detailed in Table 8.

**Table 7:** – Demographic data for Group 3

Demographic characteristics	*N* = 38
Age (years); mean (SD)	39.4 (15.9)
Age (years); median (range)	36.8 (19.2–73.8)
Female; n (%)	23 (60.5)
Proportion of participants with two ears assessed; n (%)	37 (97.4)

**Table 8:** – Hearing levels of participants in Groups 1 and 3. Hearing status based on the worst frequency (500, 1000. 2000, 4000 Hz) in the worse ear

Hearing Category	Range	Group 1 (number of participants)	Group 3 (number of participants)
Normal	≤ 25 dB HL	11	26
Mild	26–40 dB HL	5	9
Moderate	41–55 dB HL	12	2
Moderately Severe	56–70 dB HL	5	0
Severe	71–90 dB HL	5	1
Profound	≥ 91 dB HL	2	0

**Fig. 2:**
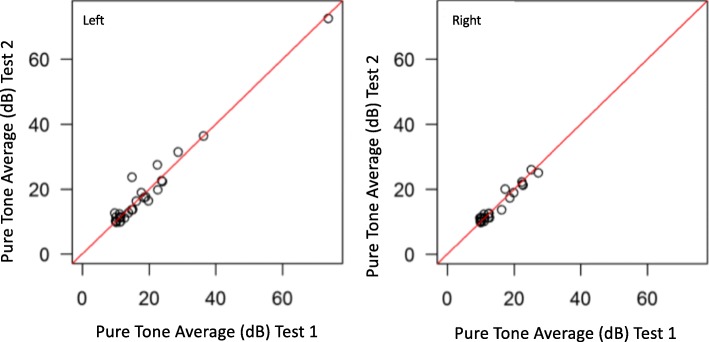
Group 3 Pure-tone threshold correlation graph for test 1 and test 2. ICC for agreement in both the left and right is 0.98

A correlation graph for test 1 and test 2 pure tone thresholds are shown in Fig. 2. The ICC for agreement between test 1 and test 2 for both left and right-ear pure tone thresholds was 0.98.

**Fig. 3:**
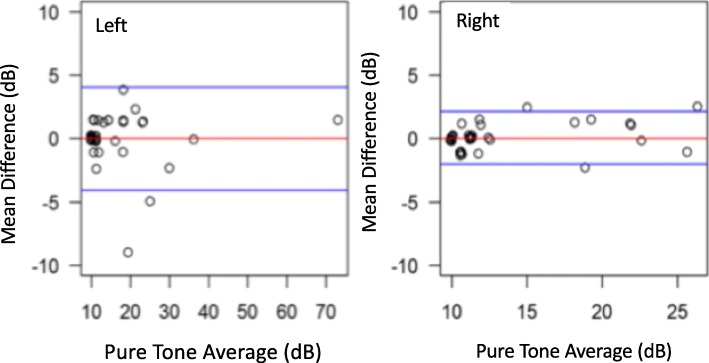
Group 3 Bland Altman Plot - Mean differences in pure tone thresholds between repeat assessments. Blue lines depict 95% limits of agreement. Mean difference of 0 (SD = 2.1) on the left, and 0.1 (SD = 1.1) on the right

For the left ear, the mean difference between repeat assessments was 0 (SD = 2.1). The 95% limits of agreement were − 4.1 dB (95% CI -5.2, − 2.9) to 4.1 dB (95% CI 2.9, 5.2). On the right side, the mean difference was 0.1 (SD = 1.1). The 95% limits of agreement were − 2 dB (95% CI -2.6, − 1.4) to 2.1 dB (95% CI 1.5, 2.8) (Fig. 3).

## Discussion

In this multi-cohort prospective study three adult cohorts underwent testing using the tablet audiometer. In Group 1, we compared the tablet audiometer to conventional audiometry. There was overall high degree of agreement between both modalities with 97% of thresholds measured within 10 dB. The impact of ambient noise in the accuracy of threshold testing has previously been described and most affects thresholds measured at 250 Hz and 8000 Hz [19, 27, 29]. Although the ambient background noise was not recorded for this study, testing was performed in a quiet clinical exam room with adjacent rooms occupied by patients attending Otolaryngology clinic. Overall, our study suggests that the tablet audiometer reported similar threshold values as traditional audiometry despite testing being performed in a non-sound insulated booth and without the coaching of a trained Audiologist. In two circumstances there was a discordance between the audiologist and tablet audiometer as to when to apply masking. The tablet audiometer is programmed to apply masking in a rule based fashion in accordance with standard audiometric practices [25]. The audiologist, in two circumstances, applied masking based on their expert opinion and experience with consideration for individual patient factors. In these scenarios the masked value was compared to the unmasked tablet audiometric threshold. Despite a discordance in the application of masking between test conditions the results suggest a high degree of agreement between tablet and conventional audiometric threshold measurements.

The definition of hearing loss that was used was meant to reflect a clinically significant hearing loss whereby a clinician may decide to offer the patient some form of intervention. We chose to characterize hearing loss by the worse ear to represent the limitations of the patient’s unaided hearing ability. As such, we used to Clinically this provides clinicians with the ability to counsel patients about how their hearing may be impacting their ability to communicate. We performed a sensitivity analysis using both 30 dB and 40 dB HL as the definition of hearing loss to enable comparisons to previous studies that have performed sensitivity and specificity analyses [19–21]. Overall, the calculated sensitivity and specificity values remained comparable. When the definition of hearing loss was lowered to 30 dB HL the sensitivity improved and the specificity decrease slightly.

Overall our results in Group 1 are similar to previous studies which have compared the tablet audiometer to conventional audiometry [19–22]. In previous studies that have evaluated the performance of other automated devices, 86–95% of tested thresholds have been shown to be within 10 dB or less compared to conventional audiometry [18, 29–32]. Thus, our results would suggest that the tablet audiometer performs at least similarly to other automated devices. Post hoc analysis revealed an important discrepancy between automated and standard audiometric methods of testing. Specifically, the automated test was set with a lower limit of 10 dB and an upper limit of 90 dB. Whereas, the clinical protocol that was used to perform conventional audiometric testing was to perform hearing threshold testing to − 10 dB. Hence, inherent differences were created by the methodology in certain instances, as a result, only frequencies where comparisons were possible for these patients were included in the statistical analyses. Future studies could mitigate this risk by aligning the protocols for tablet and traditional audiometric testing. Two additional changes were made post-hoc to our statistical analyses. As seen in Tables 2 and 3, we performed our statistical analysis for all frequencies measured as well as excluding 250 and 8000 Hz. There are several factors that may alter threshold measurements at these frequencies including ambient noise, transducer placement and tinnitus [19, 27, 29]. As a result threshold measurements at 250 Hz and 8000 Hz were removed to better represent the environment in which patients were being tested.

In Group 2, we examined the performance of the tablet audiometer to perform word recognition testing. Overall, 96.2% of scores recorded were considered equivalent. The majority (*n* = 43) of patients were found to have word recognition scores ≥80%. The high scores are expected given that the testing is completed at an optimal listening level for patients. The generalizability of the results to patients with lower word recognition scores is not clear.

This is the first study that has examined word recognition testing using the tablet audiometer. The NU-6 word list was used in both the conventional and audiometric testing. Since the tablet audiometer is an automated device the NU-6 word list was pre-recorded and played to the patient through the tablet device. Given that the word list was pre-recorded there was no opportunity to repeat the test word back to the patient in circumstances where the word was not heard due to background noise, technical issues or any other interference. Furthermore, the NU-6 word list is only available in English, monosyllabic word lists would need to be added to the software platform to facilitate inclusion patients who speak other languages.

In order to accurately compare the performance of the tablet audiometer to perform word recognition testing, the MCL established and used in the sound booth by an Audiologist was also used for the tablet audiometer testing. Additionally, the MCL should be established by someone experienced with hearing testing and the process for establishing such levels. Because the MCL is supra-threshold, it is less impacted by ambient noise therefore using the same MCL for the two test modalities is appropriate. The tablet audiometer software does allow test administrators to establish MCL for patients for general clinical use.

In Group 3, the internal reliability of the tablet audiometer was tested using a test-retest cohort design. The ICC, a measurement of internal reliability, was exceptionally high at 0.98. Moreover, the mean difference between assessments is low with narrow variance at 0 dB (SD = 2.1 dB) and 0.1 dB (SD = 1.1 dB) in left and right ears respectively, suggesting that there is minimal clinical impact in any variances between repeat assessments. A larger 95% confidence range existed for the left ear. We feel this likely reflects a learning effect given that the left ear was programmed to be tested first followed by the right ear, resulting in diminished variability for the right ear. Nonetheless, for both the left and right ear, the 95% confidence intervals were within 10db when using the portable audiometer. This is comparable to previous studies which have examined the internal reliability of conventional audiometry in healthy subjects [33].

A potential source of bias that was identified for all cohorts is the possible result of a learning effect as patients gain experience and learn the skills required to perform accurately on both conventional and tablet audiometry. Future studies could mitigate this bias by randomizing the order in which the tablet and conventional audiometric evaluations are performed.

In our opinion, the implementation of a portable self-administered tablet audiometer as a screening tool has the potential to reduce the number of referrals for conventional audiometry. Ideally, this could lead to a significant reduction in public health care costs and audiology wait times. In addition, the tablet audiometer could improve access to screening audiology, in particular, for vulnerable patient populations where access to conventional audiometry is scarce. Future studies using the tablet audiometer should aim to investigate the performance of the tablet audiometer in other clinical scenarios or patient populations such as cochlear implant transducer calibration, primary care facilities as well as tertiary care capacities, monitoring for ototoxicity in intensive-care, or oncology units. Future research is required to compare bone conduction testing as well as speech reception threshold testing between the sound booth and tablet-based audiometer.

## Conclusions

The objective of this study was to examine the validity and internal reliability of the tablet audiometer and secondarily evaluate the performance of the tablet audiometer to perform word recognition scoring. The results of this study indicate that adult audiometry and word recognition testing appears valid when performed by non-healthcare experts using a tablet audiometer outside of a sound booth in a quiet environment.

## Abbreviations

ANSI: American National Standards Institute
CASLPO: College of Audiologists and Speech-Language Pathologists of Ontario
CRC: Clinical Research Coordinator
dB HL: decibels Hearing Level
dB: Decibel
FDA: Food and Drug Administration
ICC: Intra-class correlation coefficient
MCL: Most comfortable level
NU-6: Northwestern University Word list
